# Women involvement in the informal caregiving field: A perspective review

**DOI:** 10.3389/fpsyt.2023.1113587

**Published:** 2023-01-18

**Authors:** Shyhrete Rexhaj, Alexandra Nguyen, Jérôme Favrod, Claire Coloni-Terrapon, Leslie Buisson, Anne-Laure Drainville, Debora Martinez

**Affiliations:** ^1^La Source, School of Nursing Sciences, University of Applied Sciences and Arts Western Switzerland, Lausanne, Switzerland; ^2^School of Nursing Sciences, University of Applied Sciences and Arts Western Switzerland, Fribourg, Switzerland

**Keywords:** author, informal caregiver, gender, mental disorder, women, social psychiatry

## Abstract

The patient recovery process of individual with mental health disorder is reinforced if they are connected with their community and supported by relatives. The literature has shown that caregivers are important, although their roles can lead to alterations in their own health; and women are the most involved in this role. The present review investigated women’s involvement in the informal caregiver scientific field. A literature review indicated gender differences; researchers who are women are more interested in this field than men. Even with a good representation of women in this scientific field, the results showed a statistically significant gender difference for the first and second authors, whereas there was no significant gender difference among the last authors. More efforts must be made to recognize the importance of women’s involvement in research because they raise a specific important field. Family caregivers are key players in the healthcare system, but to date, there has been little recognition of their enormous contribution. Our results also indicated the informal caregiver role is filled more by women than by men, which creates social inequalities in many domains, especially in opportunities at the professional level. Tailored interventions are required to address the specific needs and issues of family caregivers. A better redistribution of unpaid work, such as informal caregiving, compared to paid work must be made to respect gender in social existence.

## Introduction

Informal caregivers are key individuals in supporting patients with mental disorders during their recovery process. The usefulness of effective coping strategies to better manage caregiving roles has been reported in several studies; different interventions have been proposed by professionals and peer support to help informal caregivers (1, 2). Despite the effectiveness of these interventions, the health status of informal caregivers is lower than that of the general population (3), and their quality of life and well-being are affected (4). Informal caregivers’ responsibilities are not always accompanied by support focused on their needs; interventions are most often patient-centered, however new interventions tend to reduce this gap (2). Supporting a relative with mental disorder is burdensome for most informal caregivers, and the burden level is increased by several factors related to patients, households, health system facilities, personal characteristics, and community and cultural environments (5, 6). Concerning personal characteristics, women are often mentioned as primary caregivers (7). In addition, women constitute most informal caregivers for those with advanced age and mental health issues (7, 8). Although not all studies show similar results, this role can have important consequences for the mental health of female caregivers (7). Female informal caregivers were more likely to suffer from depression (51.2% vs. 38.6%, *p* = 0.031) and anxiety (51.6% vs. 38.1%, *p* = 0.020) than male informal caregivers (9). Another study investigating gender transmission of psychological distress among heterosexual and homosexual couples showed that distress transmission was stronger for women than for men (10). This finding may be explained by the pressure on women to be more emotionally involved in their relationships (10). However, emotional over-involvement could lead to negative consequences when their partners experience emotional distress (10).

As several studies have pointed out the predominant sociocultural role of women being caretakers of their relatives (7), the purpose of this article was to investigate whether this higher commitment is reflected in the scientific contributions regarding informal caregiver research. This perspective article aims through a literature review to (1) explore the gender of authors involved in caregiver research in the mental health field and (2) explore the gender of caregivers involved in the studies related to this literature review.

## Methods and results

### Scoping review

This perspective study was based on a literature review. The search was conducted on September 29, 2022, using the Web of Science core collection. The search focused on records about informal caregivers of adult psychiatric patients with the following research equation: (TS = (“mental health” OR “mental disorder*”) AND TS = (caregiv*) AND TS = (father* OR mother* OR siblings OR grandparents OR spouses) NOT ALL = (dementia OR diabetes OR cancer OR oncology OR alzheimer OR HIV OR physical)) AND (PY = = (“2022” OR “2021” OR “2020” OR “2019” OR “2018” OR “2017” OR “2016” OR “2015” OR “2014” OR “2013” OR “2012”) AND TASCA = = (“PSYCHIATRY” OR “PSYCHOLOGY DEVELOPMENTAL” OR “FAMILY STUDIES” OR “PUBLIC ENVIRONMENTAL OCCUPATIONAL HEALTH” OR “PSYCHOLOGY CLINICAL” OR “SOCIAL WORK” OR “REHABILITATION” OR “HEALTH CARE SCIENCES SERVICES” OR “HEALTH POLICY SERVICES” OR “PSYCHOLOGY” OR “NURSING” OR “PSYCHOLOGY MULTIDISCIPLINARY” OR “BEHAVIORAL SCIENCES” OR “PSYCHOLOGY SOCIAL” OR “MULTIDISCIPLINARY SCIENCES” OR “PSYCHOLOGY EXPERIMENTAL” OR “ECONOMICS” OR “ANTHROPOLOGY” OR “SOCIOLOGY” OR “COMMUNICATION” OR “SUBSTANCE ABUSE” OR “WOMEN S STUDIES” OR “ETHICS” OR “PSYCHOLOGY BIOLOGICAL” OR “SOCIAL ISSUES” OR “URBAN STUDIES” OR “DEMOGRAPHY”)). A total of 890 records were identified, and 7 supplementary records were identified from this citation search (*n* = 897). Inclusion and exclusion criteria for this selection were used by two researchers, data were processed with a bibliographic reference management software. Based on the abstract reading, all diagnostic groups were eligible, except studies focused on organic and intellectual development disorders. Studies were excluded if they focused on matters other than informal caregivers or if the informal caregivers were children. Appropriate records were screened, and 805 articles were excluded based on their titles and abstracts. The remaining 92 articles were included for gender identification by authors.

### Data analyses and results

**FIGURE 1:**
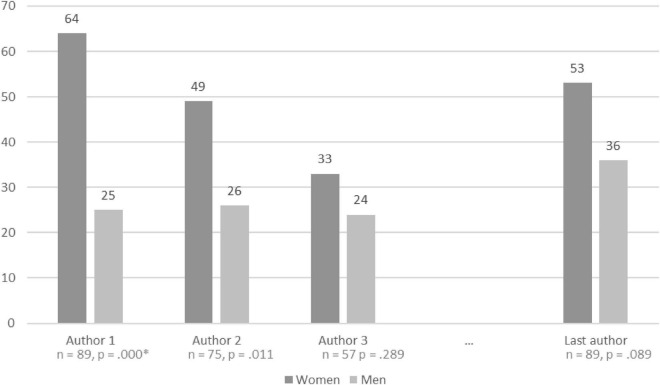
Authors’ gender distribution in the informal caregiver scientific field.

The gender of the authors (*n* = 324) were identified by 2 researchers (DM, CCT), and the data were organized into 4 categories: first, second, third, and last authors. The second author was classified as the last author when the articles were written by only 2 authors. After identifying the authors, searches were conducted on various databases to classify the authors by gender. A binary vision of gender classification, male or female, was used. Among the 4 categories, 14 authors could not be identified as either male or female. Binomial non-parametric statistical analyses were performed. Significantly more first and second authors were female than male (*n* = 89, *p* = 0.000 and *n* = 75, *p* = 0.011, respectively). No statistical significance was found between the third and last authors. The details are presented in Figure 1.

**FIGURE 2:**
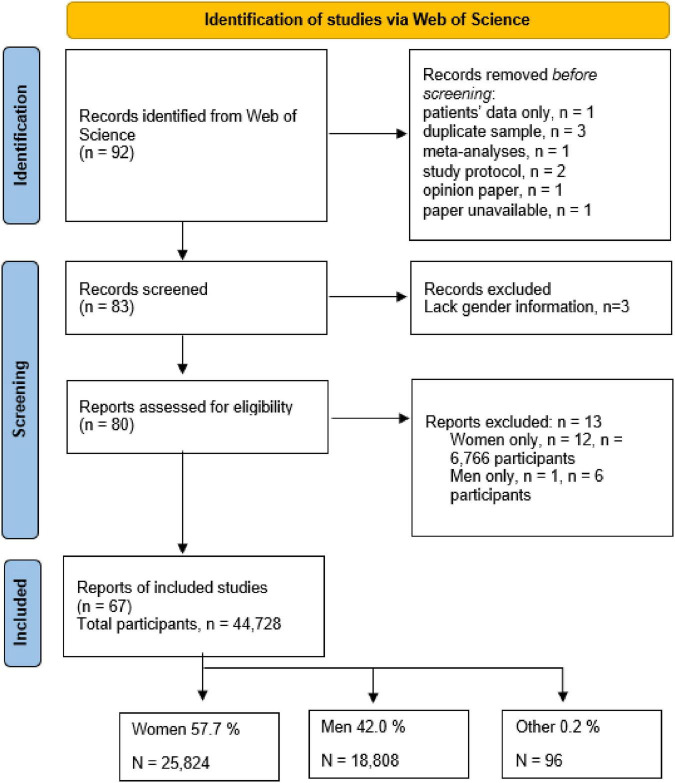
Informal caregivers’ gender in the analyzed studies. From Page et al. (30).

The second analysis concerned the proportion of women informal caregivers who participated in the studies. Articles were excluded if they were not original, unavailable, based on an identical sample, gender was not specified, or had a population other than caregivers. A total of 80 articles were included. The total sample consisted of 51,500 participants, of which 32,590 were women, 18,814 were men, and 96 were unspecified or incorrectly reported in the articles. Women represented 63.28% of the caregivers, which is consistent with previous literature. Twelve studies focused exclusively on women and 1 qualitative study focused on men. Further information is presented in Figure 2.

## Discussion

This perspective paper discusses the role of women in informal caregiving in adult psychiatry. The results indicated that women involved in research focused on the informal caregivers of people with mental disorders outnumbered men. Women, as first and second authors, were statistically more represented than men, indicating that they have a leadership role in this field. The last author is frequently considered the supervisor of the study. This result suggests that women are still under the scientific authority of men or that the last male authors seek to support the work of the women in the field. Women are frequently under the authority of men, even in this research field in which most researchers are women and women are leading authors.

One of the possible explanations for the larger percentage of women researchers’ involved in this field is probably related to the fact that women are more concerned about the informal caregiver topics (7). Social science research has highlighted the concept of gendered division of labor and this term is used by ethnologists to refer to the complementary division of labor between men and women in societies throughout history (11). The rise of materialist feminist work in the 1970s and one study by Delphy and a book by Delphy and Leonard (12, 13) focused on this gendered division of tasks and on the domestic work performed by women. They propose the notion of domestic exploitation to understand the emotional, material, symbolic, procreative, and sexual care work performed by women within the family. This line of thought allows for understanding care work as a historically and culturally embedded social practice and not as an innate role of women. Women’s interest in this issue can also be understood to stem from their socialization. Informal care is more common among women than among men, which could lead to a higher number of subjectively reported needs among women (14). Multiple roles for women have been well-documented (15). The results of one study showed that women who combined multiple roles were less depressed and had less depressive symptoms than women who only had the role of an informal caregiver (15). Even if women are believed to enjoy the informal caregiver role (16), they face diverse challenges that affect well-being and quality of life (4). To better represent men in the informal caregiving role, several studies focused on parents have included fathers, not only mothers as primary caregivers (17–19). A purposive sample is recommended to better represent a specific population. Only one study in this review presented data focused on men in a caregiving role (20), compared to 12 studies focused on women. These results suggest that informal caregivers should be identified to reduce gender discrimination.

Moreover, most of the studies focused on the “key relative” concept, which is defined as a person having a central role in a patient’s informal caregiving and considers this person has the highest burden level (14). Even if limited data of other relatives with similar burden results exist (21), all informal caregivers and cultural dimensions need to be integrated into interventions (6, 22). Men reported fewer needs while caring for a child with schizophrenia (14). Wancata et al. (14) mention “hurt pride” among men, which they define as “not accepting that an offspring suffers from a stigmatized mental disorder such as schizophrenia” as a rationale for the lower reported needs of fathers. When facing a potentially stigmatized mental disorder, men may have a more negative appraisal of mental illnesses. The importance of considering illness representations to better cope with the consequences of illnesses in daily life has been reported (23). Individuals may differ in their responses to stressful and negative events. Their coping strategies are related to their understanding of the situation and their available resources. Coping strategies are related to the environment, individual situations, and the ability to cope with stress in a specific situation, such as the coronavirus disease 2019 lockdown conditions (24).

Multiple factors need to be considered in effective interventions for supporting informal caregivers, and researchers should specifically document the sample’s characteristics to discuss the results and apply them according to gender or cultural origin (2, 25). Individualized interventions could be a good solution to support a wide range of relatives, considering their personal profiles, resources, needs, and social characteristics (2, 26). It is also recommended to propose early support, especially for young people and siblings involved in informal caregiver roles (22, 27, 28).

Informal caregivers provide more than 80% of all care in Europe and should be regarded at the political and economic levels (8). Policy effectiveness also depends on unpaid care activities to promote the well-being of the population. In Switzerland, several political projects have addressed the recognition of informal caregiver roles and appropriate support to reduce the lack of access to care and support for relatives (29). Many informal caregivers’ associations have been created over the last 30 years with the aim of offering peer support to maintain informal caregivers’ well-being and quality of life. Despite these trends, the gap between the need for care, even if policies begin to recognize their significant contribution to our welfare systems, responses of healthcare systems should target support interventions, particularly their financing. Informal caregivers’ needs will increase dramatically with the predicted shortage of healthcare professionals by 2030. In addition, gender differences in informal caregiving exacerbate women’s conditions, increase health issues for women, and especially decrease the opportunities for paid and recognized work. To promote population well-being, a redistribution of care responsibilities between men, women, the government, and the roles of informal caregivers is recommended (8).

This study has several limitations that must be considered. First, only the Web of Science database was used. This research should be replicated using more databases to increase the robustness of our results. Second, the article selection was based on the inclusion and exclusion criteria without double check or Cohen’s kappa analyses. Third, the study population focused on informal caregivers of adult psychiatric patients. Further studies should include children and adolescents who fulfill informal caregiving roles. Forth, the unavailable papers could have been acquired for reasons of completeness, however in the age of open science, they are becoming more difficult to access. In addition, unidentified gender authors could have been contacted by email to determine their gender. Finally, we used a binary vision of gender that did not reflect the existing spectrum. Further research should refine the categories to provide a classification that is more sensitive to the living experiences of individuals.

### Perspectives

Informal care should be assessed as a central activity for the balance of the healthcare system and well-being of society. It should be allocated between men and women as well as between families and governments. Moreover, equal opportunities should be offered to women and men in the workplace and at home, and a better balance between informal caregivers and professional commitments should be enabled (8).

The pioneering role of women in the scientific field of informal caregivers and the recognition of their various roles should also be highlighted for their innovative dimensions and contributions to society.

## Data availability statement

The original contributions presented in this study are included in the article, further inquiries can be directed to the corresponding author.

## Author contributions

SR, DM, and JF initiated the project, conceived the design, and performed the methods and obtained the results. DM, SR, and CC-T were involved in the literature review and data collection. DM and JF analyzed the data. DM, SR, JF, A-LD, and LB contributed to the first version of the manuscript. AN and JF critically revised the manuscript. All authors approved the final version of the manuscript.
